# Volume 100 of the *American Journal of Tropical Medicine and Hygiene*

**DOI:** 10.4269/ajtmh.18-0848

**Published:** 2019-01

**Authors:** Philip J. Rosenthal, Joseph M. Vinetz, Cathi Siegel, James W. Kazura

**Affiliations:** Authors are on the editorial board of the American Journal of Tropical Medicine and Hygiene; 1 Editor-in-Chief;; 2 Associate Editor;; 3 Managing Editor;; 4 Section Editor (former Editor-in-Chief)

This issue marks Volume 100 of the *American Journal of Tropical Medicine and Hygiene*. This milestone offers an opportunity to reflect on our *Journal*. The American Society of Tropical Medicine founded *The American Journal of Tropical Medicine* in 1921. Nearly 100 years later, the subject matter of that journal (available at http://www.ajtmh.org) is familiar, although it was influenced by the international events of the day. Among articles in the first issue were the following: “An attempt to explain the greater pathogenicity of *Plasmodium falciparum* as compared with other species,” “On the prevalence of carriers of *Endamoeba* *dysenteriae* among soldiers returned from overseas service,” and “Study of a case of yaws, contracted by an American soldier in France.” The *American Journal of Tropical Medicine and Hygiene* was formed in 1952, as a merger of the *American Journal of Tropical Medicine* and the *Journal of the National Malaria Society*, coincident with the dissolution of the National Malaria Society after the elimination of autochthonous malaria in the United States. With a name change, changes in physical appearance, recent emphasis on electronic publication, and geopolitical changes in tropical and nontropical regions of the world over the past 100 years, the focus of our *Journal* on improving global health remains steadfast.

**Figure 1.**
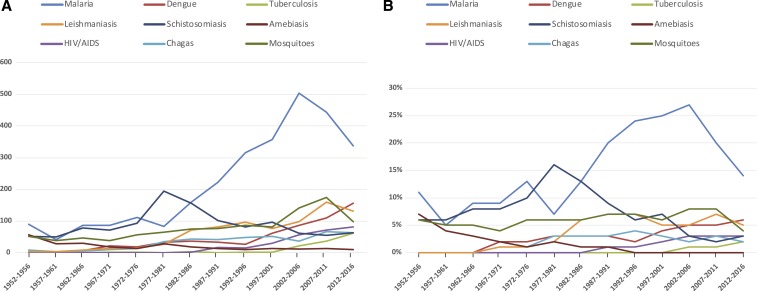
Articles published by the *Journal* on selected topics for 5-year blocks since 1952, shown as total number published (**A**) and percentage of total articles (**B**).

The first issue of the *American Journal of Tropical Medicine and Hygiene*, published in January 1952, included articles on dengue, yellow fever, scrub typhus, malaria, and amebiasis (available at http://www.ajtmh.org/content/journals/14761645/1/1). Authors included the tropical medicine giants Albert Sabin, Paul Russell, and Robert Coatney. The *Journal* has remained focused since that time on the great tropical disease problems, most of which are still with us, and has also addressed the new ones, from AIDS to Zika.

The scope of our *Journal* is broad, with articles on common parasitic, viral, and bacterial infections, and attention to many other tropical infectious diseases. Over the years the number of articles published has increased steadily, but the focus on different topics has changed, including decreased attention to schistosomiasis since about 1980, fewer articles on malaria since early in this century, and increased attention to viral infections and tuberculosis in recent years. Articles on diseases that are not infectious in etiology remain a minority, but important topics include snakebite, diabetes, and various types of poisoning. Some years ago consideration was given to removing “Hygiene” from our title, as it was suggested that this term was outdated, but in fact attention to hygiene has increased in recent years, including 19 articles published in 2017. Notably, the *Journal* is truly international. In 2017 corresponding authors were from 90 countries, and only 29% of published articles had a corresponding author from the United States.

The *Journal* has been available online since 2003; only 5% of our membership now elects to receive a print copy. Innovations in the past two decades have included the Images in Clinical Tropical Medicine section, introduced in 2008, and the Stories from the Field section, introduced in 2018. The *Journal* has published numerous supplements focused on specific tropical medicine topics. However, our main focus has not changed: our primary purpose remains to publish primary research articles pertaining to all aspects of tropical medicine and public health. Our definition of tropical medicine is broad, so articles on arthropod-borne infections and parasites transmitted outside the tropics are within our scope. Increasing numbers of submissions and an enlarging geographic scope attest to continued relevance of our *Journal* as we reach Volume 100.

The *Journal* is not only a leading international tropical medicine journal, but it also serves as the journal of the American Society of Tropical Medicine and Hygiene. As such, we encourage our members to submit important reports to the *Journal*. In addition to standard research reports, we encourage perspectives, reviews, Images in Clinical Tropical Medicine, and Stories from the Field. Our *Journal* has high impact in our fields only because our members and others offer timely and expert peer review of submitted research articles. We encourage Society members and others with interest in tropical medicine to continue to contribute their valuable insights by reviewing submitted manuscripts. These insights are essential to guide the editors in publishing the most impactful work in the *Journal*.

With Volume 100 we are proud to look back at a journal that has contributed importantly to progress in tropical medicine research and to the control of tropical disease. With continued progress, we might put ourselves out of business, as global control and elimination efforts come to fruition for one infectious disease after another. However, as the fight against tropical diseases is not easy (only one human infectious disease, smallpox, has been eradicated as of now), it seems likely that this will not occur any time soon. Also, we know that noninfectious diseases will fill any voids that arise as we conquer infectious tropical diseases, but suffer increasingly from maladies associated with an improved standard of living. So, we anticipate volumes 150 and 200, hopefully with eager future editors celebrating these milestones along with continued progress against tropical diseases. Through this changing landscape, the *American Journal of Tropical Medicine and Hygiene* will serve the membership of the American Society of Tropical Medicine and Hygiene by continuing to report on the most important advances in tropical medicine and public health.

